# Response to letter to the editor: Radiological approaches to COVID-19 pneumonia

**DOI:** 10.3906/sag-2005-230

**Published:** 2020-08-26

**Authors:** Şule AKÇAY, Tevfik ÖZLÜ, Aydın YILMAZ

**Affiliations:** 1 Department of Pulmonary Diseases, Faculty of Medicine, Başkent University, Ankara Turkey; 2 Department of Pulmonary Diseases, Faculty of Medicine, Karadeniz Technical University, Trabzon Turkey; 3 Department of Chest Diseases and Chest Surgery, University of Health Sciences, Atatürk Training and Research Hospital, Ankara Turkey

**Dear Editor,**

We would like to thank you for your interest in our review paper entitled “Radiological approaches to COVID-19 pneumonia” published in the latest issue of Turkish Journal of Medical Sciences. Principally, in this review, we aimed to discuss the approach to COVID-19 pneumonia from the perspective of a clinician. We would like to highlight the points regarding the authors’ concerns with related literature.

The authors’ first comment is that chest computed tomography (CT) indications should not be broad in COVID-19 pneumonia. We penned this review based on the experiences and observations of clinicians from various countries related with COVID-19. The definition of “suspected case” is made based on the symptoms of a patient. The role of chest CT in diagnosis of a suspected COVID-19 pneumonia case and its guiding characteristics in treatment is detailed in the outpatient clinic and emergency patient management algorithm of the Ministry of Health of the Republic of Turkey [1]. In this approach, an unenhanced chest CT is needed if the direct radiography is normal or nondiagnostic. Many pulmonologists in Turkey manage COVID-19 cases considering such algorithms according to the guidelines. Chest CT is an important diagnostic tool that strengthens the hands of pulmonologists in the diagnosis of COVID-19 pneumonia [2].

In their letter, the authors suggested that CT indication should be limited to the expected benefit that can lead to a change in the treatment or management of the patient. In accordance with this suggestion, indeed, we stated that the result of CT images might change the management and treatment of suspected COVID-19 cases, in our review.

The authors’ second comment is an objection to our interpretation that nodular appearances are atypical on both lower lobes in the first image. These appearances are not ground glass opacities. We interpreted these images as atypical, in that they are “round shaped, small nodules”, according to an expert consensus statement in the literature [3]. It is obvious that debate on definition and standardization of classifications are present in the literature. In Figure 6, while the multifocal consolidations in the right lung are more distinct, an interpretation of the existence of bilateral infiltration is made by the authors. We insist on our consideration that the infiltration in the sampled sections is unilateral.

We thank authors for the additional radiological findings provided in the third comment. These outlined issues are important radiological details in cases with COVID-19 pneumonia. We appreciate for their valuable contributions.

Visual quantitative evaluation for assessing COVID-19 burden as mentioned in the fourth comment was out of focus in our review. In our review, we did not suggest that chest CT would be used for screening purposes. Chest X-ray and chest CT are diagnostic tools for cases that meet the description of suspicious cases based on symptoms in triage (1). In a recent publication, Congliang M et al. emphasized that chest CT imaging has become the indispensable means not only in the early detection and diagnosis but also in monitoring the clinical course, evaluating the disease severity, and may be presented as an important warning signal preceding the negative RT-PCR test results [4]. As this recent paper was published after our review, we are only able to cite it in this short letter. If this result is verified with further future studies, the importance of chest CT in COVID-19 pneumonia diagnosis should be underlined even further.

However, it is a fact that, we as clinicians, have used chest CT for the diagnosis of COVID-19 pneumonia in an essential indication, and it seems that we will continue to use it in this way. Moreover, we would like to emphasize that real time polymerase chain reaction (RT- PCR) test of the nasopharyngeal and oropharyngeal swab samples is the standard diagnostic tool [5]. However, ordering CT scan for patients with suspected COVID-19 pneumonia in situations such as delaying PCR results or a false negative result in a patient with strong suspicion of disease is still a debate in literature [5,6].

Easy access of all suspicious and confirmed COVID-19 pneumonias to healthcare services and their appropriate management and early initiation of treatment, and a relatively young population explain the lower mortality rates in Turkey (2.76%, by 14th May, 2020) when compared to other countries (7). The opinions of the Fleischner Society that were mentioned in the authors’ letter are not addressed among the references in our review. Information on COVID- 19 is being revised every day based on new experiences with a multidisciplinary approach. Some of the argued opinions may be disproved by new ones in time. This is an expected situation considering the nature of scientific development. We would like to thank authors’ contributions and perspective which have enriched our manuscript.
